# Case Report: Exceptional Response to Avelumab After Failure of Electrochemotherapy in a Patient With Rapidly Progressive, PD-L1-Negative Merkel Cell Carcinoma

**DOI:** 10.3389/fonc.2021.628324

**Published:** 2021-06-17

**Authors:** Martina Torchio, Laura Cattaneo, Massimo Milione, Natalie Prinzi, Francesca Corti, Marco Ungari, Andrea Anichini, Roberta Mortarini, Antonio Occhini, Giulia Bertino, Andrea Maurichi, Jorgelina Coppa, Maria Di Bartolomeo, Filippo Guglielmo de Braud, Sara Pusceddu

**Affiliations:** ^1^ Medical Oncology Department, Fondazione IRCCS Istituto Nazionale dei Tumori, ENETS Center of Excellence, Milan, Italy; ^2^ First Pathology Division, Department of Pathology and Laboratory Medicine, Fondazione IRCCS Istituto Nazionale dei Tumori, ENETS Center of Excellence, Milan, Italy; ^3^ Department of Pathology, Azienda Socio-Sanitaria Territoriale di Cremona, Cremona, Italy; ^4^ Human Tumor Immunobiology Unit, Department of Research, Fondazione IRCCS Istituto Nazionale dei Tumori, ENETS Center of Excellence, Milan, Italy; ^5^ Department of Otolaryngology Head and Neck Surgery, Fondazione IRCCS Policlinico San Matteo, Pavia, Italy; ^6^ Melanoma and Sarcoma Surgical Unit, Fondazione IRCCS Istituto Nazionale dei Tumori, ENETS Center of Excellence, Milan, Italy; ^7^ GI and Liver Transplantation Surgical Unit, Fondazione IRCCS Istituto Nazionale dei Tumori, ENETS Center of Excellence, Milan, Italy; ^8^ Oncology and Hematology-Oncology Department, University of Milan, Milan, Italy

**Keywords:** skin neoplasms, immunotherapy, electrochemotherapy, MCC, avelumab, Merkel cell carcinoma, ECT, case report

## Abstract

This case report shows, for the first time, a patient experiencing a complete response after one dose of avelumab following extensive disease progression with prior electrochemotherapy (ECT) treatment. We suggest that ECT may help to establish a tumor microenvironment favorable to immunotherapy. Merkel cell carcinoma (MCC) is a highly aggressive skin cancer with seldom durable chemotherapy responses. ECT has recently emerged as a potential treatment option for several malignancies, including MCC. Avelumab, an anti-programmed cell death-ligand 1 (PD-L1) monoclonal antibody, became the first approved treatment for patients with metastatic MCC. ECT has been shown to activate the immune response, but it is still unknown how ECT may affect patient’s response to subsequent immunotherapy. We report a case of a patient with MCC who presented with a rapidly growing skin nodule of the right cheek and experienced extensive disease progression following surgical debulking and ECT treatment. The patient received a flat dose of 800 mg avelumab intravenously every 2 weeks showing complete tumor regression after only one dose. Immunohistochemical analysis of surgical and post-ECT biopsies collected from the primary lesion revealed tumor expression of programmed cell death protein-1 (PD-1), but not PD-L1. Analysis of the tumor samples also revealed no expression of Merkel cell polyomavirus (MCPyV). Comparison of the biopsies showed a decrease in myeloid and T-cell markers after ECT but an increase in major histocompatibility complex (MHC) class I expression on tumor cells. Additionally, the patient experienced an increase in neutrophil-to-lymphocyte ratio and lactate dehydrogenase values post-ECT, which subsequently decreased with avelumab treatment. As of 30 October 2019, the patient was still receiving avelumab treatment and had an ongoing complete response. In this case report, a patient with PD-L1-negative and MCPyV-negative MCC who had disease progression following ECT experienced complete tumor regression with avelumab treatment, suggesting, for the first time to our knowledge, that ECT may help to establish a tumor microenvironment favorable to immunotherapy *via* a potential abscopal effect. Tumor-intrinsic PD-1 expression and modulation of MHC class I antigens after ECT may contribute to the clinical efficacy of avelumab in this context.

## Introduction

Merkel cell carcinoma (MCC) is a rare and highly aggressive neuroendocrine tumor associated with clonal integration of the Merkel cell polyomavirus (MCPyV), ultraviolet (UV) radiation exposure, advanced age, and immunosuppression (1, 2). Approximately 65% of patients with MCC present with local disease, and approximately 26 and 8% of patients present with nodal and distant metastatic disease, respectively (3).

MCC can grow rapidly, and treatment options are limited; the current standard of care for patients with localized MCC is surgery with or without adjuvant radiation therapy (1). Although MCC is considered chemo-sensitive, responses to chemotherapy are rarely durable; median overall survival with chemotherapy is approximately 10 months (1, 2). Recently, emerging evidence suggests that electrochemotherapy (ECT; electrical pulses administered alongside chemotherapy) may be an effective treatment option for patients with MCC, although published literature is limited to case reports (4–6). Furthermore, immune checkpoint inhibitors (ICIs), including the anti-programmed cell death-ligand 1 (PD-L1) monoclonal antibody avelumab, are currently recommended for the treatment of patients with metastatic MCC (mMCC) based on promising results including durable responses observed in clinical trials (1, 7–9).

Here, we report the case of an 80-year-old man who presented with rapidly growing skin nodule of the right cheek. A surgical debulking was performed and the microscopical histopathological examination showed small cells with a round-oval nucleus and scarce cytoplasm. Immunohistochemical analysis results were consistent with MCC. The postsurgical physical examination revealed an irregular purplish lesion near the right preauricular region, close to the surgical scar. Computed tomography (CT) of the face, neck, chest, and abdomen revealed malignant disease in the preauricular region but showed no distant metastases.

After surgical debulking in January 2019, the patient experienced relapse in April 2019. He then initiated ECT without results since the lesion increased in size. When the lesion reached dimensions of 8.5 × 10 cm, the patient started avelumab therapy.

After receiving one dose of avelumab, the patient experienced a complete response following extensive disease progression with prior ECT treatment. To our knowledge, this has never been documented in literature before.

## Materials and Method

Ki-67, synaptophisin, chromogranin-A, TTF1, CK7, CK AE1-AE3, PD-1, PD-L1, MHC class I, HLA-DR, CD14, CD68, CD163, CD3, CD4, CD8, Granzyme B, and CD20, were investigated by immunohistochemistry (IHC). Briefly, sections 2.5/3-µm thick were cut from paraffin blocks, dried, de-waxed, rehydrated, and unmasked (with Dako PT-link, EnVision™ FLEX Target Retrieval Solution, High/Low pH). Antibodies were incubated with a commercially available detection kit (EnVision™ FLEX+, Dako, Denmark) in an automated Immunostainer (Dako Immunostainer Link 48). IHC for PD-L1 were made using Ventana Benchmark Ultra IHC/ISH System immunostainer (Ventana Medical Systems, Tucson, AZ, USA) following manufacturer instructions. Antibody dilutions, clones, and specifics are reported in detail in **Table 1**.

**Table 1:** Antibody sources and dilutions.

Antigens	Dilution	Code Number	Clone	Source
KI-67 (M)	1/400	M7240	Mib-1	Dako, Agilent, Denmark
Synaptophisin (M)	1/200	M7315	Dak-Synap	Dako, Agilent, Denmark
Chromogranin-A (M)	1/100	M0869	Dak-A3	Dako, Agilent, Denmark
TTF1(M)	1/2000	M3575	8G7G3	Dako, Agilent, Denmark
Cytokeratin 7 (M)	1/200	M7018	OV-TL	Dako, Agilent, Denmark
Cytokeratin (M)	1/100	M3515	AE1/AE3	Dako, Agilent, Denmark
PD-1 (M)	1/50	ab52587	NAT105	Abcam
PD-L1 (M)	Prediluted	740-4859	SP142	Ventana Medical System-Roche
MHC class I (M)	1/4000	ab6405	OX18	Abcam
HLA-DR(M)	1/500	MS-133-P0	LN3	Thermo Fisher Scientific
CD14 (M)	1/500	ab133335	EPR 3653	Abcam
CD68 (M)	1/3000	M0814	KP1	Dako, Agilent, Denmark
CD163 (M)	1/200	NCL-L-CD163	10D6	Leica Biosystems
CD3 (P)	1/400	A0452	Polyclonal	Dako, Agilent, Denmark
CD4 (M)	1/300	M7310	4B12	Dako, Agilent, Denmark
CD8 (M)	1/20	M7103	C8/144B	Dako, Agilent, Denmark
Granzyme B (M)	1/50	M7235	GrB-7	Dako,Agilent, Denmark
CD20	1/400	M0755	L26	Dako,Agilent, Denmark

M, Monoclonal; P, Polyclonal; TTF-1, Thyroid transcription factor-1.

The expression of inflammatory markers (PD-1, PD-L1, MHC class I, HLA-DR, CD14, CD68, CD163, CD3, CD4, CD8, Granzyme B, and CD20) on tumor cells and within the tumor microenvironment was evaluated using a semiquantitative scoring system according to a previous analysis by Milione and colleagues (10). Scoring considered the staining intensity (I) in a three-tiered scale (1, less intense than the control; 2, intensity superimposable to the control according to the manufacturer indications; 3, more intense than the control). Extension (E) was defined as the percentage of positive cells for each marker (0, 0%; 1, <25%; 2, 25–50%; 3, 51–74%; 4, 75–100%). A final score was determined from the product of I E.

## Case Description

In January 2019, an 80-year-old man, came to the physician’s attention due to the appearance of a nodular, fixed lesion of 1.5 × 1.5 cm in dimension, localized in the right cheek. The lesion appeared around 3 months before (October 2018) he came to the hospital and was initially interpreted as a cystic lesion. The patient reported preauricular pruritus, sense of tension, and sporadic pain during chewing. In January 2019, due to the preauricular localization of the lesion and its infiltrative features with suspected highly aggressive cutaneous characteristics, the clinicians proposed a surgical debulking with a radical intention. At the time of the surgical proposal, clinical conditions were suitable with chronological age, with an Eastern Cooperative Oncology Group (ECOG) performance status score of 1. In anamnesis, the patient presented grade 1 arterial hypertension (controlled with medical chronic therapy), mild grade 1 hypercholesterolemia, mild cognitive impairment, and non-clinically significant mitral valve insufficiency (patient performed annual cardiologic follow-up).

Familial anamnesis was negative for neoplastic disorders. The patient’s history excluded both professional exposure to neoplastic risk factors or any other patient-dependent risk factors, such as smoking habits, or ethnicity. The patient’s history did not present features suspicious for hereditary/syndromic familial presentation of the neoplastic event.

**Figure 1:**
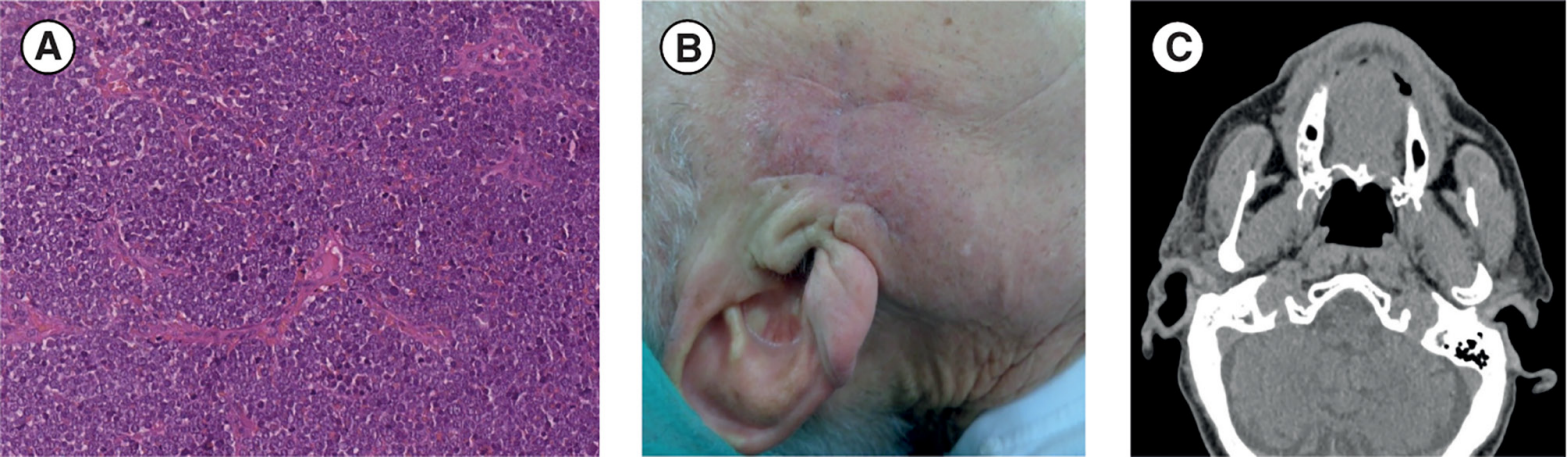
**(A)** Diagnosis: histological image of hematoxylin and eosin. section (scale bar: 50 µm) shows small tumor cells with a round-oval nucleus and poor cytoplasm that are very densely arranged in a diffuse pattern of growth. **(B, C)** Post-debulking surgery restaging: **(B)** Post-debulking clinical presentation with a purplish lesion (approximately 1.5 × 1.5 cm) situated near the right preauricular region close to the surgical scar. **(C)** Face and neck CT scan (axial projection) showing residual disease in the right preauricular region. CT, computed tomography.

After surgical debulking of the lesion (in January 2019), the patient presented localized pain associated with persistence of homolateral hearing loss and homolateral (right) preauricular sensitivity deficit. Microscopical histopathological examination showed small cells with a round–oval nucleus and scarce cytoplasm. IHC staining confirmed the expression of cytokeratin (CK)20, CK7, chromogranin A, synaptophysin, and high levels of Ki67 (80%), whereas thyroid transcription factor 1 (TTF1) was not expressed; these IHC results are consistent with MCC (11). A diagnosis of MCC extended at the surgical resection margin was confirmed (**Figure 1A**). Additionally, MCPyV was not present. After 2 months, the patient returned for postsurgical restaging and a physical examination, which revealed an irregular purplish lesion of approximately 1.5 × 1.5 cm situated near the right preauricular region, close to the surgical scar (**Figure 1B**). CT of the face, neck, chest, and abdomen revealed malignant disease in the preauricular region (**Figure 1C**), including two pseudonodular areas, but showed no distant metastases.

At disease relapse, in April 2019, the patient presented progressive increment of pain and loss of appetite due to chew-related pain. Together with dimensional increment of the lesion, discomfort worsened in terms of sense of tension in preauricular and later cervical areas, and loss of appetite. The patient began ECT, consisting of an intravenous (IV) bolus infusion of bleomycin 15,000 IU/m^2^ administered 8 min before delivery of electroporation by means of hexagonal array electrodes (5,000 Hz) connected to an electric pulse generator (Cliniporator; IGEA Clinical Biophysics; Carpi, Italy). In June 2019 (at the first post-ECT assessment), the treated lesion had increased in size (3.5 × 4.5 cm). Subsequently, the patient received regular follow-ups in order to distinguish whether the increase in lesion size was due to postprocedural inflammation or to disease progression.

**Figure 2:**
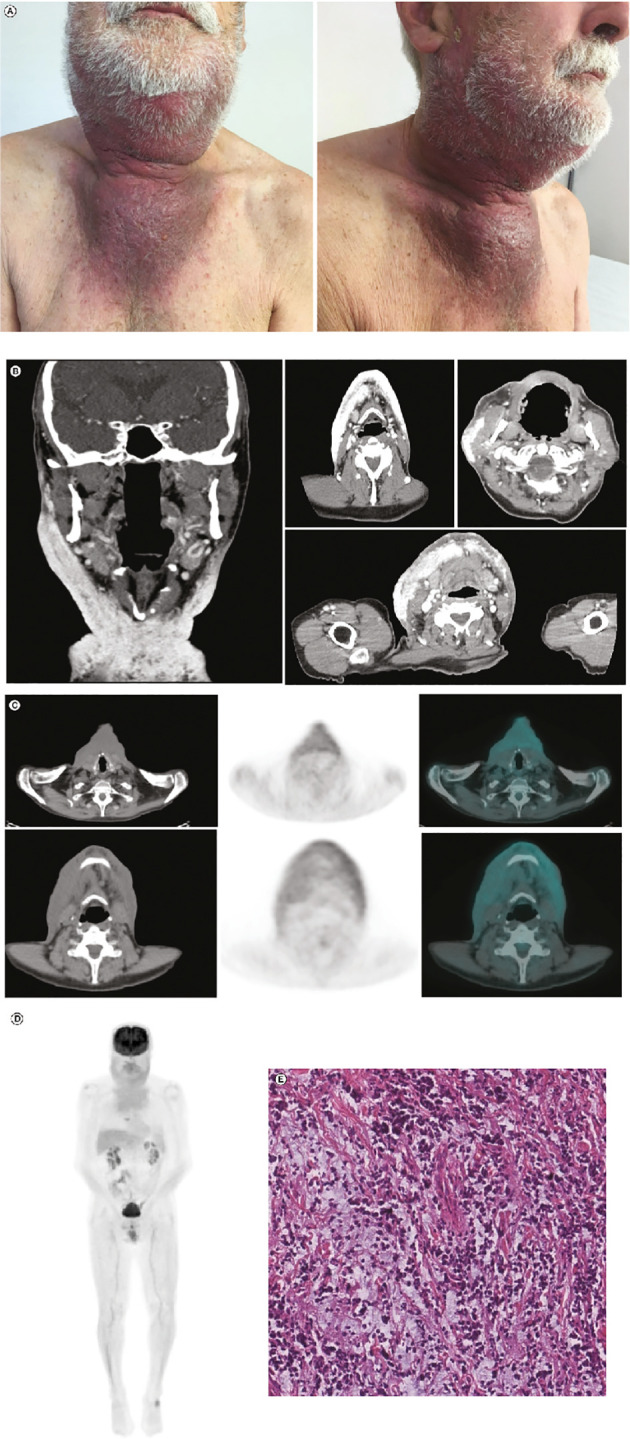
Post-ECT restaging: **(A)** Post-ECT clinical presentation with an extensive, dark lesion (approximately 8.5 × 10.0 cm) in the preauricular and laterocervical regions. **(B)** Coronal (leftmost panel) and axial (right panels) CT scans showing extensive infiltrates in the subcutaneous tissues of the bilateral laterocervical region and right preauricular area. **(C)** Left to right: CT scan, FDG-PET scan, and CT and FDG-PET fusion images showing pathological accumulation in the preauricular area extending until the bilateral laterocervical and sternal level. **(D)** Total-body FDG-PET image confirming significant FDG uptake in mandibular, laterocervical, and sternal regions. **(E)** Hematoxylin and eosin stain (scale bar: 50 µm) of the tumor sample taken after ECT. Compared with the tumor sample collected before ECT, there was a decrease in neoplastic cellularity and edematous stroma. CT, computed tomography; ECT, electrochemotherapy; FDG-PET, 18-fluorodesossyglucose-positron emission tomography; MCC, Merkel cell carcinoma.

At the end of June 2019, the lesion measured 5.0 × 7.0 cm and was erythematous. After 1 week (at the beginning of July 2019), the erythematous lesion had increased substantially in size (8.5 × 10 cm) in the preauricular region and extended to the lateral cervical region (**Figure 2A**). In July 2019, together with the symptoms experienced in April 2019, the patient reported pain and pruritus in lateral cervical area due to tumor cutaneous infiltration. CT scans confirmed disease progression and revealed extensive infiltrates, including in the pseudonodular areas, in the subcutaneous tissues of the bilateral lateral cervical region, and in the right preauricular area (**Figure 2B**); 18-fluorodesossyglucose-positron emission tomography (FDG-PET) confirmed pathological accumulation in the preauricular area extending to the bilateral lateral cervical and sternal level (**Figure 2C**), as confirmed by the whole body PET-FDG **(** **Figure 2D**). Additionally, biopsy and IHC staining of the preauricular cutaneous area showed a high-grade neuroendocrine tumor, confirming progression (**Figure 2E**).

**Figure 3:**
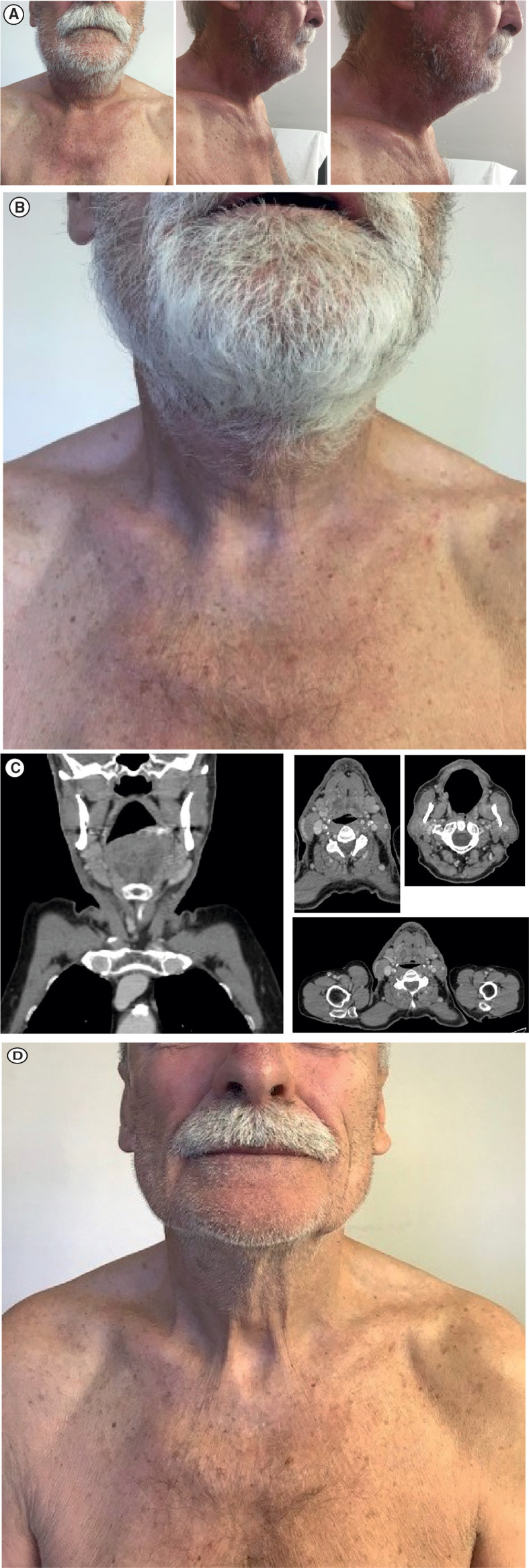
Response to avelumab treatment: **(A)** Clinical presentation of substantial measurable reduction in lesion size after one dose of avelumab. **(B)** Clinical presentation of confirmed complete regression of the tumor mass after three doses of avelumab. **(C)** Face and neck coronal (leftmost panel) and axial (right panels) CT scans showing complete radiological response. **(D)** Clinical presentation of ongoing complete response on 30 October 2019. CT, computed tomography.

Given the extensive progression, a decision was made to start a regimen of avelumab flat dose 800 mg IV every 2 weeks in August 2019; at this time, the lesion was 13.0 × 15.0 cm. After the first administration of avelumab, a substantial reduction in lesion size was observed with no measurable lesion remaining (**Figure 3A**). After three administrations of avelumab, a complete response was confirmed according to iRECIST (modified Response Evaluation Criteria in Solid Tumors guideline for immunotherapy) (12) (**Figures 3B, C**). As of 30 October 2019, the patient was still continuing with avelumab flat dose 800 mg IV every 2 weeks, with CT scans every 3 months; he had an ongoing complete response, with no evidence of new lesions or progressive disease (**Figure 3D**).

**Figure 4:**
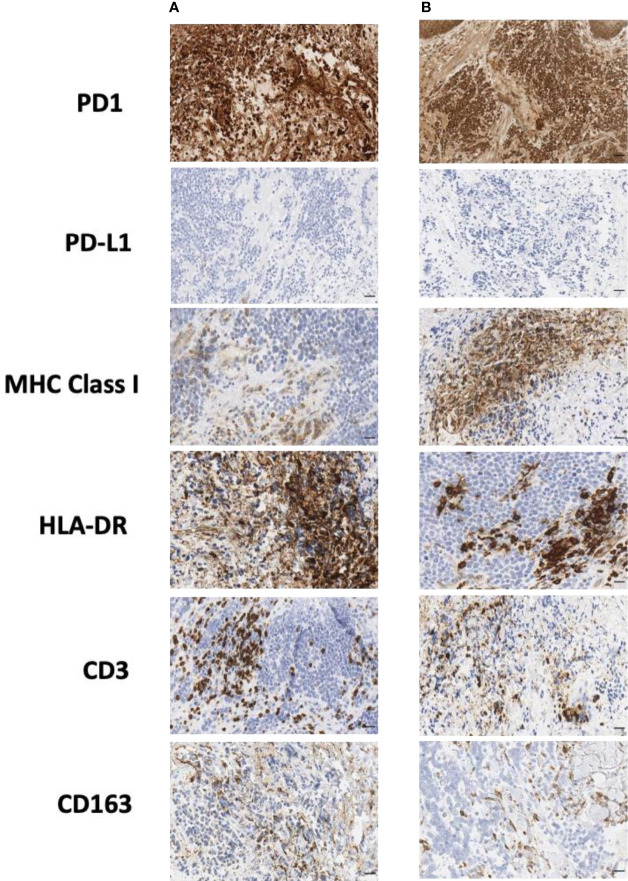
Immunohistochemical staining of biopsy samples taken **(A)** during surgery and **(B)** after ECT. CD, cluster of differentiation; ECT, electrochemotherapy; HLA, human lymphocyte antigen; MHC, major histocompatibility complex; PD-1, programmed cell death protein-1; PD-L1, programmed cell death-ligand 1. Scale bar: 50 µm.

In order to better understand the immune profile of the tumor microenvironment and its relationship with tumor cell features, a comparative IHC analysis was performed in two tumor samples, respectively obtained during debulking surgery and after ECT. The final scores obtained from the product of I × E, as described in the *Material and Methods* section, are summarized in **Table 2**. **Figure 4** shows the images related to the most important data resulting from immunohistochemical analysis performed on bioptic samples at diagnosis **(Column A)** and after ECT **(Column B).**

**Table 2:** Expression of inflammatory markers using a semiquantitative scoring system that considers staining intensity (I) on a three-tiered scale (1, less intense than control; 2, intensity superimposable to control according to manufacturer indications; 3, more intense than control), as well as extension (E), defined as the percentage of positive cells for each marker (0, 0%; 1, <25%; 2, 25–50%; 3, 51–74%; 4, 75–100%) (10). Total score = I E.

Marker (score)	Surgical biopsy		Post-ECT biopsy	
	*Expressed on tumor cells*	*Expressed in tumor microenvironment*	*Expressed on tumor cells*	*Expressed in tumor microenvironment*
PD-1	9	0	6	0
PD-L1	0	0	0	0
MHC class I	2	4	4	0
HLA-DR	2	4	2	0
CD14	0	2	0	2
CD68	0	4	0	2
CD163	0	4	0	2
CD3	0	6	0	1
CD4	0	6	0	2
CD8	0	2	0	2
Granzyme B	0	2	0	0
CD20	0	2	0	2

ECT, electrochemotherapy; HLA, human lymphocyte antigen; MHC, major histocompatibility complex; PD-1, programmed cell death protein-1; PD-L1, programmed cell death-ligand 1.

Interestingly, in both the surgical and post-ECT samples, programmed cell death protein-1 (PD-1) was found to be expressed at a low level on tumor cells, and PD-L1 was not expressed either on tumor cells or within the tumor microenvironment. Additionally, fewer myeloid cells were present in the post-ECT sample compared with the surgical sample, as shown by the decrease in CD68 and CD163 expression in the tumor microenvironment. Major histocompatibility complex (MHC) class I antigen expression on tumor cells increased in the post-ECT sample compared with the surgical sample, while CD3 and CD4 markers in the tumor microenvironment substantially decreased.

The absolute count of neutrophils and lymphocytes and lactate dehydrogenase (LDH) serum values were collected at each visit and after each ECT or avelumab treatment. The derived peripheral blood neutrophil-to-lymphocyte ratio (NLR) was also evaluated. With these data, we aimed to correlate blood-toxicities or complications and to investigate the potential prognostic significance and/or predictive value of NLR or LDH markers on treatment response.

At first diagnosis (January 2019), the basal NLR value was 2.45, and LDH was 256 U/L. These values increased after ECT in parallel with disease progression and increased tumor size until August 2019. The patient then showed a reduction in NLR and LDH values concurrent with achieving a complete response after the first dose of avelumab (September 2019); these values continued to decrease after three cycles of avelumab, when complete response was radiologically confirmed (**Supplementary Table 1** and **Supplementary Figure 1**). As of October 2020, NLR and LDH values were continuing to decrease.

## Timeline

See **Supplementary Table 2** for the timeline.

## Diagnostic Assessment

In this study, the following diagnostic tests were performed: physical examination, IHC, neutrophil count, CT of the head, neck, thorax, and abdomen, and FDG-PET. The primary diagnostic challenge was to evaluate the immune-context of the patient before treatment with avelumab. The diagnosis in October 2018 was of MCC extended at the surgical resection margin.

## Discussion

Incidences of MCC have increased in recent years; approximately 5,000 new cases of MCC occur each year in the USA and Europe (13, 14). MCC is associated with clonal integration of the MCPyV (approximately 80% of cases) or UV radiation exposure and commonly occurs in patients who are elderly and fair skinned (11). MCC usually presents as a rapidly growing purple nodule situated in the upper region of the body, including the shoulders, head, and neck. Diagnosis of MCC is dependent on IHC staining patterns, including the expression of CK20 and a lack of TTF1 (11). Most patients with MCC present with local disease, for which surgery with or without concomitant radiotherapy is the current recommended treatment (1). Unfortunately, disease progression can occur quickly, and recurrence is common; approximately one-third of patients with MCC develop distant metastases (1).

For patients with mMCC, current guidelines recommend enrollment in a clinical trial or systemic therapy with an ICI (1). In 2017, avelumab became the first approved treatment for mMCC based on the results of the JAVELIN Merkel 200 trial (7, 9). Avelumab binds to PD-L1, preventing its interaction with PD-1 and subsequent T-cell exhaustion (15). Durable responses have been observed with first- and second-line or later avelumab in patients with mMCC. In part A of JAVELIN Merkel 200, 88 patients with mMCC and progressive disease after chemotherapy received avelumab. After ≥36 months of follow-up, the objective response rate (ORR) was 33.3%, including complete responses in 11.4%; median duration of response was 40.5 months (16). In part B, 116 patients with mMCC who were naive to systemic therapy received avelumab; after a median follow-up of 21.2 months, the ORR was 39.7%, and 30.2% of patients had a response that lasted ≥6 months (17). Another ICI, pembrolizumab (anti-PD-1 antibody), has shown encouraging results in patients with advanced MCC. In the KEYNOTE-017 trial, 50 patients with stage IIIB or IV MCC who were naive to systemic therapy received pembrolizumab, and after a median follow-up of 14.9 months, the ORR was 56.0%, including complete responses in 24.0% (8). In this case report, treatment with avelumab led to a complete response that was ongoing as of 10 April 2020.

Recently, ECT has emerged as a potential treatment option for MCC. ECT allows the delivery of non-permeant chemotherapy, such as bleomycin, into cells through administration of short, intense electric pulses that increase cell membrane permeability, thereby enhancing the cytotoxic activity of chemotherapy (4). ECT, most commonly used with bleomycin, is well tolerated and has been shown to be an effective treatment strategy in several tumor types, including non-melanoma skin cancers (4). Complete responses have been reported with ECT in some patients with MCC; however, published literature remains limited (4–6). In the case presented here, ECT was chosen due to the small area of local relapse and absence of distant lesions; previous clinical data described the efficacy of ECT in small and locally relapsed MCC lesions of head and neck origin (5, 6). Additionally, the location of the lesion (preauricular region) and its proximity to the surgical scar meant that treatment with radiotherapy had an increased risk of radiotherapy-related toxicities and the potential to be less effective due to altered vascularization in the area. However, in this patient, treatment with ECT was followed by extensive disease progression, highlighting the need for further research into the use of ECT in patients with MCC.

Preclinical data has shown that ECT activates the immune system, can induce immunogenic cell death, and may lead to an abscopal effect, wherein an antitumor response is elicited outside the primary target of treatment (18). However, little is known about how the immunogenic mechanisms elicited by ECT may affect patient response to subsequent immunotherapy.

In the present case report, IHC analysis of surgical and post-ECT biopsies indicated a substantial remodeling of the immune contexture after ECT. In particular, we observed decreased expression of T-cell and myeloid-cell markers, but an increase in tumor cell expression of MHC class I antigens. The latter suggests that antigen presentation by tumor cells increased after ECT, which may have improved tumor recognition by the reactivated adaptive immune system following subsequent avelumab treatment. Furthermore, a post-ECT reduction in infiltrating T cells may not have compromised the efficacy of anti-PD-L1 treatment, consistent with recent analyses of T-cell clonotype differences before and after anti-PD-1 therapy in basal cell carcinoma or squamous cell carcinoma (19) and in the context of neoadjuvant PD-1 blockade in melanoma (20). Results of these studies suggest that the antitumor T cells reactivated by ICI treatment are not the exhausted T cells already present in pretherapy lesions but are instead a newly recruited population of T cells. Additionally, the reduction in T-cell markers observed in the post-ECT sample contrasted with previous evidence indicating that ECT promotes CD3+ and CD8+ T-cell infiltration of treated lesions (21). A potential explanation for our findings may be the extensive disease progression observed after ECT; the rapidly growing tumor may have outpaced the ability of the immune system to maintain infiltrating T cells at the level observed at surgical excision of the primary lesion.

Of note, in this patient, PD-1 was expressed on a subset of tumor cells. As shown initially in melanoma, tumor cell-intrinsic PD-1 expression may foster tumor growth by activation of the mechanistic target of rapamycin kinase (mTOR) signaling pathway upon binding to PD-L1 (22). Therefore, in this case, interruption of the PD-1/PD-L1 axis by avelumab may have counteracted potential protumoral signaling by PD-1. However, a recent study has shown that, in immunodeficient murine models, engagement of the PD-1/PD-L1 axis due to coexpression of both PD-1 and PD-L1 on tumor cells can suppress tumor growth due to inhibition of the protein kinase B (AKT) and extracellular signal-regulated kinase (ERK) pathways (23). Blockade of the PD-1/PD-L1 interaction in this model promotes tumor growth in the absence of adaptive immunity. These contrasting findings suggest that the biological functions of the PD-1/PD-L1 axis, when receptor and ligand are both expressed on tumor cells, may be dependent on tumor context, and that engagement or interruption of this interaction may have different effects depending on the specific tumor setting where immunotherapy targeting PD-1 or PD-L1 is employed.

The use of PD-L1 and MCPyV as predictive biomarkers in MCC has not yet been established. In the case reported here, the patient’s tumor was both PD-L1-negative and MCPyV-negative, and an exceptional complete response was achieved with avelumab after ECT. However, this PD-L1-negative result may be due to heterogeneous expression of PD-L1 within the tumor. Similarly, responses to avelumab have also been observed, irrespective of PD-L1 or MCPyV status, in patients with mMCC enrolled in the phase II JAVELIN Merkel 200 trial (16), suggesting that these biomarkers may have limited utility to predict a response to ICI treatment.

In recent years, the potential prognostic significance of NLR and LDH levels in patients with advanced tumors has been investigated (24, 25); however, the role of NLR and LDH levels remains unknown for patients with MCC treated with immunotherapy. Neutrophilia indicates a systemic inflammatory response, whereas lymphopenia has been associated with impaired cell-mediated immunity; elevated pretreatment NLR is associated with poorer prognosis in advanced tumors (24). In the case reported here, although the patient had elevated NLR and LDH values prior to avelumab treatment, a rapid decrease was observed, concurrent with complete tumor response. These findings suggest that the treatment sequence of ECT followed by immunotherapy alters the inflammatory markers present in tumor cells, as well as in peripheral blood. Further studies are needed to determine the roles of NLR and LDH as potential biomarkers to consider when selecting patients for immunotherapy treatment (26).

## Patient Perspective

Already after the first administration of avelumab, the patient reported sudden regression of sense of tension, pain, and also conditioning loss of appetite. After the third administration of avelumab, the patient reported complete symptom regression. Since the start of avelumab, the patient reported optimal tolerance without significative toxicities and presented objective features of amelioration: cutaneous and subcutaneous grade 2 later cervical erythema, which was documented before starting the systemic treatment, macroscopically reduced after the first administration, and completely disappeared after three cycles.

The patient provided consent to the use of his data for the present case report.

## Conclusion

In conclusion, we report an exceptional complete response in a patient with PD-L1–negative and MCPyV-negative MCC after relatively few administrations of avelumab and following extensive disease progression with ECT treatment. This case report, although inherently anecdotal in its nature, suggests that ICIs are a potential therapeutic option for patients with MCC who are non-responsive to ECT and that the treatment sequence of ECT followed by immunotherapy may improve clinical outcome. More data are needed to identify how immunogenic mechanisms after ECT may affect response to subsequent ICI treatment. However, the findings reported here suggest that promotion of antigen presentation, changes in inflammatory markers, and perhaps interruption of tumor-intrinsic PD-1/PD-L1 signaling may contribute to the efficacy of avelumab treatment.

## Data Availability Statement

The original contributions presented in the study are included in the article/**Supplementary Material**. Further inquiries can be directed to the corresponding author.

## Ethics Statement

Ethical review and approval were not required for the study on human participants in accordance with the local legislation and institutional requirements. The patients/participants provided their written informed consent to participate in this study. Written informed consent was obtained from the individual(s) for the publication of any potentially identifiable images or data included in this article.

## Author Contributions

MT participated in the clinical management of the patient and contributed to data analysis and interpretation, paper writing, manuscript editing, and final approval. LC, MM, MU, and AA contributed to the histological analysis, data analysis and interpretation, manuscript writing, editing, and approval. NP and FC participated in the clinical management of the patient, data analysis and interpretation, and manuscript editing and approval. RM contributed to the histological analysis, data analysis and interpretation, paper writing, editing, and approval. AO, GB, AM, JC, MB, FB, and SP participated in the clinical management of the patient, data analysis and interpretation, and manuscript editing and approval. All authors contributed to the article and approved the submitted version.

## Conflict of Interest

MT reports receiving non-financial support from Merck KGaA. LC reports receiving non-financial support from Merck KGaA. FC reports receiving non-financial support from Merck KGaA. AA reports receiving honoraria and research funding from Bristol Myers Squibb. FB reports receiving honoraria from Amgen, AstraZeneca, Bayer, Bristol Myers Squib, Celgene, Daiichi Sankyo, Dephaforum, Eli Lilly, Ignyta, Incyte, Instituto Gentili, Menarini, Merck & Co., Novartis, OCTIMET Oncology, Pfizer, Pharma Research, Pierre Fabre, Roche, Servier, and Tiziana Life Sciences. SP reports receiving honoraria and research funding from Ipsen and Pfizer; honoraria from AAA Pharmaceutical, Italfarmaco, and Novartis; and non-financial support from Merck KGaA. NP reports receiving non-financial support from Merck KGaA.

The remaining authors declare that the research was conducted in the absence of any commercial or financial relationships that could be construed as a potential conflict of interest.

## Abbreviations

CD, cluster of differentiation; CK, cytokeratin; CT, computed tomography; ECOG, Eastern Cooperative Oncology Group; ECT, electrochemotherapy; FDG-PET, 18-fluorodesossyglucose-positron emission tomography; HLA, human lymphocyte antigen; ICI, immune checkpoint inhibitor; IHC, immunohistochemistry; iRECIST, Response Evaluation Criteria in Solid Tumors guideline for immunotherapy; IV, intravenous; LDH, lactate dehydrogenase; MCC, Merkel cell carcinoma; MCPyV, Merkel cell polyomavirus; MHC, major histocompatibility complex; mMCC, metastatic Merkel cell carcinoma; mTOR, mechanistic target of rapamycin kinase; NLR, peripheral blood neutrophil-to-lymphocyte ratio; ORR, objective response rate; PD-1, programmed cell death protein-1; PD-L1, programmed cell death-ligand 1; TTF1, thyroid transcription factor 1; UV, ultraviolet.
