# Supplementary material for: Case Report: Exceptional Response to Avelumab After Failure of Electrochemotherapy in a Patient With Rapidly Progressive, PD-L1-Negative Merkel Cell Carcinoma
Source: Front Oncol. 2021 Jun 17;11:628324. doi: 10.3389/fonc.2021.628324 (PMC8248546; doi:10.3389/fonc.2021.628324)
Supplement: Supplementary file 1 [file DataSheet_1.pdf]

CARE case report guidelines

CARE Checklist of information to include when writing a case report

(cc) BY-NC-ND

Topic	Item	Checklist item description	Reported on Line
Title	1	The diagnosis or intervention of primary focus followed by the words "case report"	Title
Key Words	2	2 to 5 key words that identify diagnoses or interventions in this case report, including "case report"	26-27
Abstract (no references)	3a	Introduction: What is unique about this case and what does it add to the scientific literature?	30-32
	3b	Main symptoms and/or important clinical findings	42-44
	3c	The main diagnoses, therapeutic interventions, and outcomes	43-53
	3d	Conclusion—What is the main "take-away" lesson(s) from this case?	54-59
Introduction	4	One or two paragraphs summarizing why this case is unique (**may include references**)	83-85
Patient Information	5a	De-identified patient specific information.	87-88
	5b	Primary concerns and symptoms of the patient.	90-95
	5c	Medical, family, and psycho-social history including relevant genetic information	88-90
	5d	Relevant past interventions with outcomes	90-92
Clinical Findings	6	Describe significant physical examination (PE) and important clinical findings.	96-100
Timeline	7	Historical and current information from this episode of care organized as a timeline	155
Diagnostic Assessment	8a	Diagnostic testing (such as PE, laboratory testing, imaging, surveys).	157-158
	8b	Diagnostic challenges (such as access to testing, financial, or cultural)	158-159
	8c	Diagnosis (including other diagnoses considered)	159-160
	8d	Prognosis (such as staging in oncology) where applicable	NA
Therapeutic Intervention	9a	Types of therapeutic intervention (such as pharmacologic, surgical, preventive, self-care)	101, 117, 122-123
	9b	Administration of therapeutic intervention (such as dosage, strength, duration)	101-103, 117-118, 122-124
	9c	Changes in therapeutic intervention (with rationale)	101-117, 118-124
Follow-up and Outcomes	10a	Clinician and patient-assessed outcomes (if available)	NA
	10b	Important follow-up diagnostic and other test results	96-100, 104-107, 108-118, 122-124
	10c	Intervention adherence and tolerability (How was this assessed?)	NA
	10d	Adverse and unanticipated events	NA
Discussion	11a	A scientific discussion of the strengths AND limitations associated with this case report	234-237, 246-250
	11b	Discussion of the relevant medical literature **with references**.	162-204
	11c	The scientific rationale for any conclusions (including assessment of possible causes)	206-233
	11d	The primary "take-away" lessons of this case report (without references) in a one paragraph conclusion	255-263
Patient Perspective	12	The patient should share their perspective in one to two paragraphs on the treatment(s) they received	NA
Informed Consent	13	Did the patient give informed consent? Please provide if requested	Yes [x] No [ ]
