# Supplementary material for: Case Report: Exceptional Response to Avelumab After Failure of Electrochemotherapy in a Patient With Rapidly Progressive, PD-L1-Negative Merkel Cell Carcinoma
Source: Front Oncol. 2021 Jun 17;11:628324. doi: 10.3389/fonc.2021.628324 (PMC8248546; doi:10.3389/fonc.2021.628324)

**Supplementary Material**

**Supplementary Table 1. Immuno-serological analysis of NLR and LDH serum levels.**

| **Treatment stage (date)** | **Tumor size** | | **NLR** | **LDH (U/L)** |
| --- | --- | --- | --- | --- |
|  | ***Dimensions (cm)*** | ***Approximate area (cm^2^)*** |  |  |
| Diagnosis (January 2019) | 3.0 × 4.0 | 12 | 2.45 | 256 |
| Relapse after surgery (March 2019) | 1.5 × 1.5 | 2 | 2.01 | 269 |
| Before ECT (April 2019) | 3.0 × 3.0 | 9 | 1.97 | 278 |
| After ECT (early June 2019) | 3.5 × 4.5 | 16 | 2.74 | 284 |
| After ECT (early July 2019) | 8.5 × 10.0 | 85 | 3.88 | 302 |
| Before avelumab (August 2019) | 13.0 × 15.0 | 195 | 3.91 | 310 |
| Complete response after first dose of avelumab (September 2019) | 0 | 0 | 2.78 | 332 |
| Ongoing complete response after third dose of avelumab (October 2019) | 0 | 0 | 2.64 | 297 |
| Ongoing complete response with continued avelumab (December 2019) | 0 | 0 | 1.84 | 273 |
| Ongoing complete response with continued avelumab (February 2020) | 0 | 0 | 1.68 | 261 |
| Ongoing complete response with continued avelumab (April 2020) | 0 | 0 | 1.02 | 180 |

ECT, electrochemotherapy; LDH, lactose dehydrogenase; NLR, peripheral blood neutrophil-to-lymphocyte ratio.

**Supplementary Table 2. Timeline.**

| **Date** | **Relevant data** |
| --- | --- |
| October 2018 | The patient was an 80-year-old man with a rapidly growing skin nodule of the right cheek. He had an Eastern Cooperative Oncology Group (ECOG) performance score of 1 and a history of arterial hypertension, hypercholesterolemia, mild cognitive impairment and non-clinically significant mitral valve insufficiency |
| January 2019 | Surgical debulking of the lesion. Microscopical histopathological examination showed small cells with a round–oval nucleus and scarce cytoplasm. Immunohistochemistry staining confirmed the expression of cytokeratin (CK)20, CK7, chromogranin A, synaptophysin and high levels of Ki67 (80%), whereas thyroid transcription factor 1 (TTF1) was not expressed. MCPyV was not present |
| March 2019 | Physical examination revealed an irregular purplish lesion of approximately 1.5 × 1.5 cm situated near the right preauricular region, close to the surgical scar. Computed tomography (CT) of the face, neck, chest and abdomen revealed malignant disease in the preauricular region, including two pseudonodular areas, but showed no distant metastases |
| April 2019 | ECT begins: intravenous (IV) bolus infusion of bleomycin 15,000 IU/m^2^ administered 8 minutes before delivery of electroporation by means of hexagonal array electrodes (5000 Hz) connected to an electric pulse generator |
| June 2019 | In June 2019, the treated lesion had increased in size (3.5 × 4.5 cm) |
| Last week of June 2019 | The lesion measured 5.0 × 7.0 cm and was erythematous |
| Early July 2019 | The erythematous lesion had increased substantially in size (8.5 × 10 cm) in the preauricular region and extended to the lateral cervical region. CT scans confirmed disease progression and revealed extensive infiltrates, including in the pseudonodular areas, in the subcutaneous tissues of the bilateral lateral cervical region, and in the right preauricular area |
| August 2019 | The lesion was 13.0 × 15.0 cm. Start of a regimen of flat dose 800 mg IV every 2 weeks |
| 2 weeks later | Substantial reduction in lesion size with no measurable lesion remaining |
| September 2019 | A complete response was confirmed according to iRECIST |
| 30 October 2019 | The patient was continuing with avelumab flat dose 800 mg IV every 2 weeks, with CT scans every 3 months; he had an ongoing complete response, with no evidence of new lesions or progressive disease. |

**Supplementary Figure 1.** **NLR and LDH serum values over time, from diagnosis to the last recorded administration of avelumab (April 2020).** ECT, electrochemotherapy; NLR, peripheral blood neutrophil/lymphocyte ratio; LDH, lactate dehydrogenase.


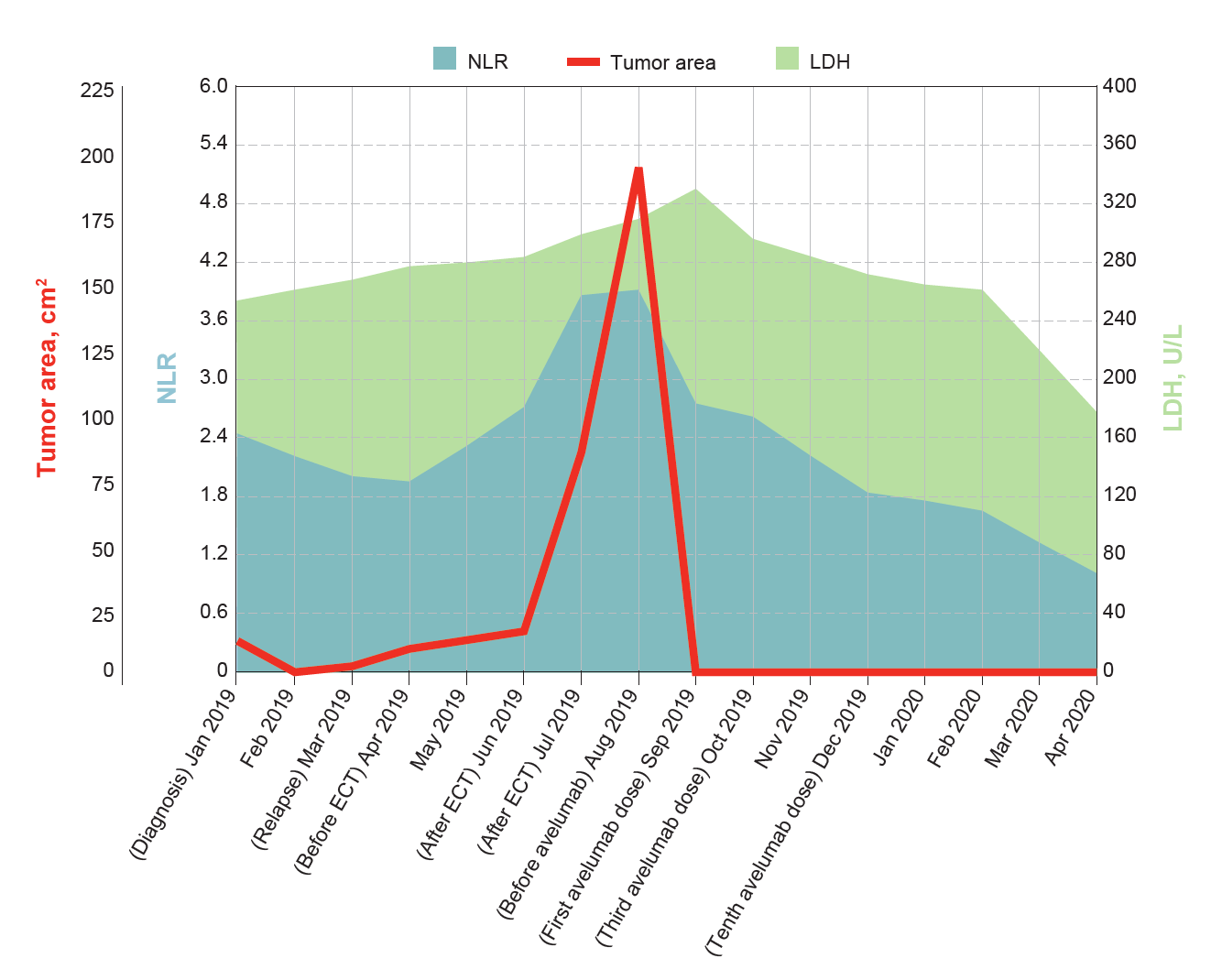

Supplement: Supplementary file 2 [file DataSheet_2.docx]
